# The MADS-Box Transcription Factor *EjAGL65* Controls Loquat Flesh Lignification *via* Direct Transcriptional Inhibition of *EjMYB8*

**DOI:** 10.3389/fpls.2021.652959

**Published:** 2021-04-07

**Authors:** Hang Ge, Yan-na Shi, Meng-xue Zhang, Xian Li, Xue-ren Yin, Kun-song Chen

**Affiliations:** ^1^Zhejiang Provincial Key Laboratory of Biometrology and Inspection & Quarantine, College of Life Sciences, China Jiliang University, Hangzhou, China; ^2^Zhejiang Provincial Key Laboratory of Horticultural Plant Integrative Biology, Zhejiang University, Zijingang Campus, Hangzhou, China; ^3^The State Agriculture Ministry Laboratory of Horticultural Plant Growth, Development and Quality Improvement, Zhejiang University, Zijingang Campus, Hangzhou, China

**Keywords:** loquat, MADS, MYB, chilling injury, lignin biosynthesis, Mδ subgroup

## Abstract

Loquat fruit accumulates lignin in its flesh when undergoing chilling injury during postharvest storage, making it a suitable model for the study of flesh lignification. Transcriptional regulation of lignin biosynthesis is principally controlled by the NAC-MYB transcriptional cascade in model plants. Previous research has demonstrated that EjMYB8 activates lignin biosynthesis through direct interaction with the promoter of *Ej4CL1*. However, the classic NAC-MYB gene regulation network has not been established. Here, the MADS-box gene *EjAGL65* was discovered by screening a cDNA library using the *EjMYB8* promoter as bait in yeast. A phylogenetic analysis and structural comparisons revealed that *EjAGL65* belongs to the Mδ subgroup of the MADS-box family, whose members have not been reported as being involved in the regulation of lignin deposition. *EjAGL65* transcription was downregulated at 0°C compared to 5°C, indicating a negative correlation with the change of lignin content. A dual-luciferase assay indicated that *EjAGL65* is capable of inhibiting the promoter activity of *EjMYB8 in vivo*. These results showed that the Mδ MADS-box gene *EjAGL65* transcriptionally regulates *EjMYB8* during postharvest chilling induced flesh lignification, which differs from the classical regulation model of lignin biosynthesis that has been illustrated for developmental lignin accumulation.

## Introduction

Lignin is a pivotal component of vascular tissues that enabled plants to conquer dry land by reinforcing cell walls. In most cases, a lack of lignin may trigger severe defects in plant development. For example, disruption of lignin biosynthesis results in dwarfism in *Arabidopsis* (Im Kim et al., 2014) and crops, such as rice, suffer lodging due to low lignin deposition in stems (Peng et al., 2014). Although lignin is important for plant growth and development, it may negatively affect the quality of plant products. Many studies have attempted to alleviate the lignification progress in edible plant organs such as fruit (Dangcham et al., 2008; Cao et al., 2010; Suo et al., 2018). Loquat is a fruit common in China that is sensitive to low temperature. During postharvest storage and transportation, the flesh of loquat fruit readily lignifies if injured by chilling or mechanical force, which limits the distance the fruit can be transported. Lignified flesh becomes less juicy and hard to chew, resulting in the deterioration of fruit quality (Jin et al., 2014; Cao et al., 2018). Due to the negative impact on the postharvest storage of loquat, the molecular mechanism underlying flesh lignification must be determined.

Lignin deposition is a complicated process that includes monolignol synthesis and polymerization. Monolignols are synthesized from phenylalanine *via* the phenylpropanoid pathway in the cytoplasm before they are transported to the apoplast where they undergo oxidation before incorporation into the lignin polymer. The amino group of phenylalanine is first removed by phenylalanine ammonia-lyase (PAL), and *p*-coumaric acid is generated by adding a hydroxyl group to the C_4_ position of the aromatic ring by cinnamate 4-hydroxylase (C4H). The *p*-coumaric acid is then catalyzed sequentially by 4-coumarate: coenzyme A (CoA) ligase (4CL), *p*-hydroxycinnamoyl-CoA: quinate shikimate *p*-hydroxycinnamoyltransferase (HCT), *p*-coumarate 3-hydroxylase (C3H), caffeoyl-CoA *O*-methyltransferase (CCoAOMT), cinnamoyl-CoA reductase (CCR), and ferulate 5-hydroxylase (F5H), caffeic acid *O*-methyltransferase (COMT), which results in methyl oxidation in the aromatic ring at the C_3_ (coniferaldehyde) site or both the C_3_ and C_5_ (sinapaldehyde) sites. The *p*-coumaraldehyde can only be converted by 4CL and CCR using *p*-coumaric acid as the substrate. Finally, the products of aldehydes are reduced to alcohols by cinnamyl alcohol dehydrogenase (CAD), generating *p*-coumaryl alcohol, coniferyl alcohol, and sinapyl alcohol (Boerjan et al., 2003; Vanholme et al., 2019). These monomers are oxidized/radicalized by peroxidase (PRX) and laccase (LAC), followed by polymerization *via* oxidative coupling. Several genes encoding key lignin biosynthetic enzymes, such as *Ej4CL1* (Li et al., 2017) and *EjCAD5* (Xu et al., 2019b), have been identified and play a role in loquat flesh lignification; *Ej4CL1* encodes 4CL, which catalyzes *p*-coumaric acid to *p*-coumaroyl-CoA. *EjCAD5* encodes CAD, which converts *p*-coumaraldehyde, coniferaldehyde, and sinapaldehyde to their alcohol forms. Transcript abundance of these genes increases under chilling injury, which is in accordance with the higher lignin content and increased fruit firmness. Nevertheless, it is unknown how these enzymes are precisely regulated.

Flesh lignification is intensely regulated at the transcriptional level. Some evidence has been found indicating the involvement of various transcription factor families in the regulation of lignin biosynthesis. According to previous studies, ectopic expression of MYB (Xu et al., 2014b; Wang et al., 2016), NAC (NAM, ATAF1, ATAF2, and CUC2; Ge et al., 2017), APETALA2/Ethylene Response Factor (AP2/ERF; Zeng et al., 2015; Zhang et al., 2020), Heat Shock Factor (HSF; Zeng et al., 2016), and Homobox from *Arabidopsis thaliana* (HAT; Xu et al., 2019b) family members in loquat flesh resulted in changes in the expression of phenylpropanoid pathway genes. For example, the transcripts of *Ej4CL1* can be induced by EjERF39 and EjHSF1 or repressed by EjAP2-1 and EjbHLH1, resulting in changes in the lignin content (Zeng et al., 2015, 2016; Xu et al., 2019b; Zhang et al., 2020). EjNAC3 and EjHAT1 can modify the expression of *EjCAD*-like and *EjCAD5*, respectively (Ge et al., 2017; Xu et al., 2019b). Although these transcription factors may be regulators of the phenylpropanoid pathway, most cannot physically interact with promoters of target genes on their own, indicating that mediators are required.

Recent publications have shown that MYB family proteins are mediators of most critical regulators of flesh lignification (Kamdee et al., 2014; Zhang et al., 2016). Through binding to the AC elements located in the promoter region, MYB family members provide other transcription factors with physical routes by which they can regulate the transcripts of *Ej4CL1*. For example, by protein interaction with EjMYB1, EjAP2-1 suppresses the EjMYB1-induced expression of *Ej4CL1* (Zeng et al., 2015). Similarly, EjbHLH1 inhibits the transcription of *Ej4CL1* by forming a protein complex with EjMYB2 (Xu et al., 2019a). In addition, the transcripts of *Ej4CL1* that increased due to *EjMYB8* could be further induced by the synergism between EjERF39 and EjMYB8 (Zhang et al., 2020). Thus, lignin biosynthesis can be regulated by protein-protein interactions with MYBs, which is a strategy frequently adopted by transcription factors that cannot directly bind to promoters of genes in the phenylpropanoid pathway.

Despite the numerous studies conducted on manipulating the biological functions of MYB members at the protein-protein level, limited research has been conducted on proteins that transcriptionally regulate MYB genes during flesh lignification. It is widely accepted that NAC domain proteins play a role in the development of the secondary cell wall by affecting transcript abundance of multiple MYB genes (Boerjan et al., 2003; Li et al., 2015; Yang et al., 2017). For instance, *AtMYB58* and *AtMYB63* directly control the monolignol biosynthesis gene *At4CL1*, and *AtMYB58* and *AtMYB63* are both regulated by the secondary cell wall master switch gene AtSND1, which represents a part of the complex NAC-MYB cascade (Zhou et al., 2009). Although several lignification-related NAC family members have been characterized in the loquat lignification process, interactions between NACs and MYBs have not yet been identified. EjNAC3 has been found to directly bind to *EjCAD*-like instead of forming a NAC-MYB cascade (Ge et al., 2017). The findings from studies on loquat support the hypothesis that the regulation of stress induced lignin deposition differs from that of developmental lignin (Cesarino, 2019). Though common regulators are employed, such as MYB and NAC genes, there may be novel regulators involved in regulating stress induced lignification. Therefore, further information is needed on partners that interact with *EjMYBs* to form a transcriptional cascade.

MADS-box genes constitute an important group of transcriptional regulators that have been characterized as regulators of plant morphology (Yu, et al., 2014), flower development (Pelaz et al., 2000), flowering time (Michaels and Amasino, 1999), fruit development, and ripening (Gimenez et al., 2010; Martel et al., 2011). Generally, MADS-box genes are rarely considered as regulators of lignin deposition. Although a thinner pericarp with altered lignin content was observed in tomato fruit with a silenced *TAGL1* MADS-box gene (Gimenez et al., 2010), the detailed mechanism whereby MADS-box genes regulate components of the secondary cell wall remains unclear.

In the present study, the upstream regulators of a potent activator of lignin, EjMYB8, were investigated, with the aim of discovering the cascade regulation model. Using yeast one-hybrid screening, a MADS-box domain protein EjAGL65 was discovered to physically interact with the promoter region of *EjMYB8*. According to phylogenetic analyses, EjAGL65 was the most similar to AtAGL65, which belongs to the Mδ subgroup of the MADS-box family. Transcript abundance of *EjAGL65* was monitored at 0 and 5°C, two treatments that promote and alleviate lignification, respectively. The expression pattern of *EjAGL65* showed a negative correlation with lignin content and fruit firmness. A dual-luciferase assay confirmed that EjAGL65 inhibited the activity of both *EjMYB8* and the *Ej4CL1* promoter. These results revealed the involvement of MADS-box genes and the novel MADS-MYB transcriptional cascade in fruit flesh lignification.

## Materials and Methods

### Plant Materials and Treatments

Loquat fruits from *Eriobotrya japonica* Lindl. cultivar “LYQ” were collected in Luqiao, Zhejiang Province, China. The fruits and treatments were previously described in Xu et al. (2014b). Generally, the fruits were selected for uniformity and divided into two batches with approximately 150 fruits in each batch. One batch was kept at 5°C, while the other at 0°C, as the control. Fruits were sampled at 0, 1, 2, 4, and 6 days of storage. At each time point, 15 fruits with the pericarp and seeds removed were sampled; therefore, there were three mixed flesh groups containing five fruits each as three biological replicates. The collected flesh samples were immediately frozen in liquid nitrogen and stored at −80°C until use.

### Yeast One-Hybrid Screening and Confirmation

Yeast one-hybrid screening was performed using the Matchmaker Gold Yeast One-Hybrid Library Screening System (Clontech, Mountain View, CA, United States). The promoter of *EjMYB8* was isolated using the GenomeWalker universal kit (Clontech) then inserted into the pAbAi vector. Primers used for genome walking and pAbAi construction are listed in Supplementary Tables S1, S2, respectively. The recombinant *EjMYB8*-pAbAi vector was linearized and transformed into a Y1HGold yeast strain. After testing for autoactivation, 100 ng/ml Aureobasidin A (AbA) was used for library screening (Supplementary Figure S1). The Y1HGold[*EjMYB8*/AbAi] yeast strain was transfected together with the cDNA library, which was previously constructed by Zhang et al. (2016). After 5 days of growth at 30°C, every individual yeast colony was picked for PCR analysis and then sequenced by Huajin Company (Shanghai, China). The obtained cDNA fragments were used for an *in silico* search for annotations using the Basic Local Alignment Search Tool.^1^ Five putative transcription factors were selected for further confirmation (listed in Supplementary Table S3). Primers designed for full-length CDS isolation and construction of pGADT7 vectors were listed in Supplementary Tables S4, S5, respectively. The obtained sequences were uploaded to the NCBI database (GenBank numbers are given in Supplementary Table S6).

To confirm true positive interactions, we separately transformed pGADT7 vectors with the genes listed in Supplementary Table S5 into the Y1HGold [*EjMYB8*/pAbAi] yeast strain. Empty pGADT7 plasmids were transformed as the negative control. Transformed yeast was grown on SD/-Leu medium containing 100 ng/ml of AbA.

### Phylogenetic Analysis

Additional nine MADS-box genes were added to the previously reported 107 MADS-box genes by searching the TAIR database.^2^ A total of 116 *Arabidopsis* and two Populus MADS-box genes were used in the phylogenetic analysis (Supplementary Table S7). The analysis was completed by the ClustalX v1.81. The protein sequence of EjAGL65 was aligned with 116 MADS-box genes using the default settings of gap open and gap extension cost, and the Blosum 30 was selected as the protein weight matrix. A phylogenetic tree was constructed using the neighbor-joining (NJ) method, and the bootstrap value was set to 1,000. Results of the alignments were visualized using Figtree v1.4.4.^3^

### RNA Extraction and cDNA Synthesis

The “LYQ” flesh samples collected at each time point were used for total RNA extraction following the method described by Shan et al. (2008). Potential genomic DNA contamination was eliminated using the TURBO DNA-free kit (Ambion, Austin, TX, United States). The quality and quantity of freshly extracted RNA were determined by gel electrophoresis and spectrophotometry, respectively (Implen, Westlake Village, CA, United States). A total of 1 μg DNA-free RNA were used to synthesize first-strand cDNA using the iScript cDNA Synthesis Kit (Bio-Rad, Hercules, CA, United States). A 10-fold dilution of cDNA was made prior to the real-time PCR analysis.

### Real-Time PCR Analysis

Real-time PCR was carried out using the CFX96 Real-Time System (Bio-Rad) with SsoFast EvaGreen Supermix (Bio-Rad). The reaction mixture contained 10 μl SYBR PCR supermix, 6 μl diethylpyrocarbonate-treated H_2_O, 2 μl diluted cDNA template, and 1 μl of each primer (10 μM). Primers used for real-time PCR were designed using Primer3 (version 4.0.0) and are listed in Supplementary Table S8.^4^ The primers were tested to ensure their specificity for unique genes (Yin et al., 2010). The real-time PCR program was set as follows: a pre-denaturation step of 95°C for 30 s, followed by for 45 cycles of 95°C for 10 s and 60°C for 10 s. A melting curve analysis was also conducted. Three biological replicates were collected from the data from each time point.

### Dual-Luciferase Assay

Dual-luciferase assays were performed as described previously (Min et al., 2014). Full-length *EjAGL65* genes were amplified with the primers listed in Supplementary Table S9 and integrated into the pGreen II 0029 62-SK vector (SK). The promoter of *EjMYB1*, *EjMYB2*, and *EjMYB8* was individually inserted into the pGreen II 0800-LUC vector (LUC). The LUC vectors containing promoters of lignin biosynthesis genes in *Arabidopsis* were constructed by Xu et al. (2014b).

All of the recombinant SK and LUC vectors were transfected into *Agrobacterium tumefaciens* GV3101 and stored as glycerol stocks. Transfected *Agrobacterium* cultures were grown on LB plates containing 50 μg/ml kanamycin and 25 μg/ml gentamycin for 2 days, then restreaked on new LB plates and grown for 1 day. *Agrobacterium* cultures were suspended in infiltration buffer (10 mM MES, 10 mM MgCl_2_, 150 mM acetosyringone, pH 5.6) to optimal density (OD_600_ = 0.75), then 1 ml *Agrobacterium* culture containing transcription factors was mixed with 100 μl *Agrobacterium* containing promoters. Finally, the mixtures were injected into tobacco leaves with needleless syringes. Three days after infiltration, the fluorescence intensity of LUC and REN were measured using dual luciferase assay reagents (Promega, Madison, WI, United States). Five replicates were conducted for each transcription factor and promoter combination.

### Statistical Analysis

OriginPro 2020 software (Microcal Software Inc., Northampton, MA, United States) was used to perform statistical analyses and to draw figures. Student’s *t*-tests were conducted on data from luciferase assays; differences were considered significant at 5%. One-way ANOVA followed by means comparison using a Tukey’s test was applied for comparing data from real-time PCR at the 5% confidence level. The average value and SE of three replicates were calculated using Excel 2017 (Microsoft, Seattle, WA, United States).

## Results

### Screening Potential Targets of *EjMYB8*

**Figure 1:**
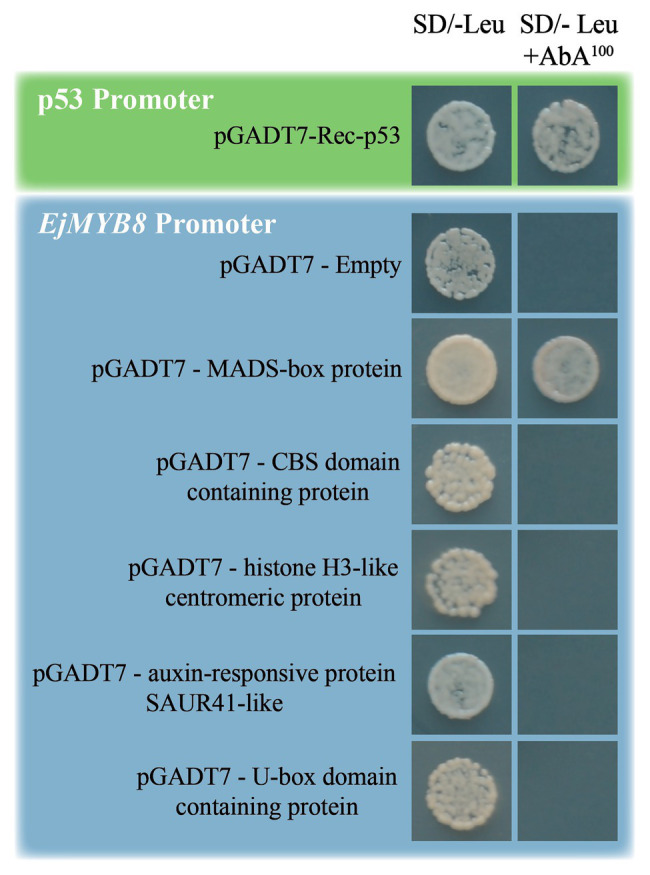
A yeast one-hybrid analysis was used to identify a MADS-box protein, which binds the *EjMYB8* promoter. Sequences of five genes were inserted individually into the pGADT7 vector then transformed into Y1HGold[pro-*EjMYB8*] for an *in vivo* Protein-DNA binding assay based on the results of the yeast one-hybrid library screening. Growth of yeast on SD/-Leu plates with 100 ng/ml Aureobasidin A (AbA) indicates that the interaction is genuinely positive. The promoter of p53 was also transformed into yeast as the positive control. The negative control was an empty pGADT7 vector.

Previous research has demonstrated that NAC and MYB genes mediate lignification in loquat flesh (Xu et al., 2014b), but the classic NAC-MYB transcription cascade has not been previously revealed. Here, we chose to identify the upstream regulators of *EjMYB8* because of its significance in cold triggered expression by yeast one-hybrid screening (Wang et al., 2016). The promoter region of *EjMYB8* was integrated into the Y1HGold yeast strain, and we performed a screening under the stress of 100 ng/ml AbA to eliminate the background growth of yeast. Following the amplification of library fragments by yeast colony PCR, a total of 32 colonies were sequenced and annotated (listed in Supplementary Table S10). We chose five putative transcription factors for further confirmation. Consequently, a MADS-box domain-containing protein (GenBank No. MF942415) was identified as having a positive interaction with the promoter of *EjMYB8*; the other four proteins had false-positive results (Figure 1).

### Phylogenetic Analysis of EjAGL65

**Figure 2:**
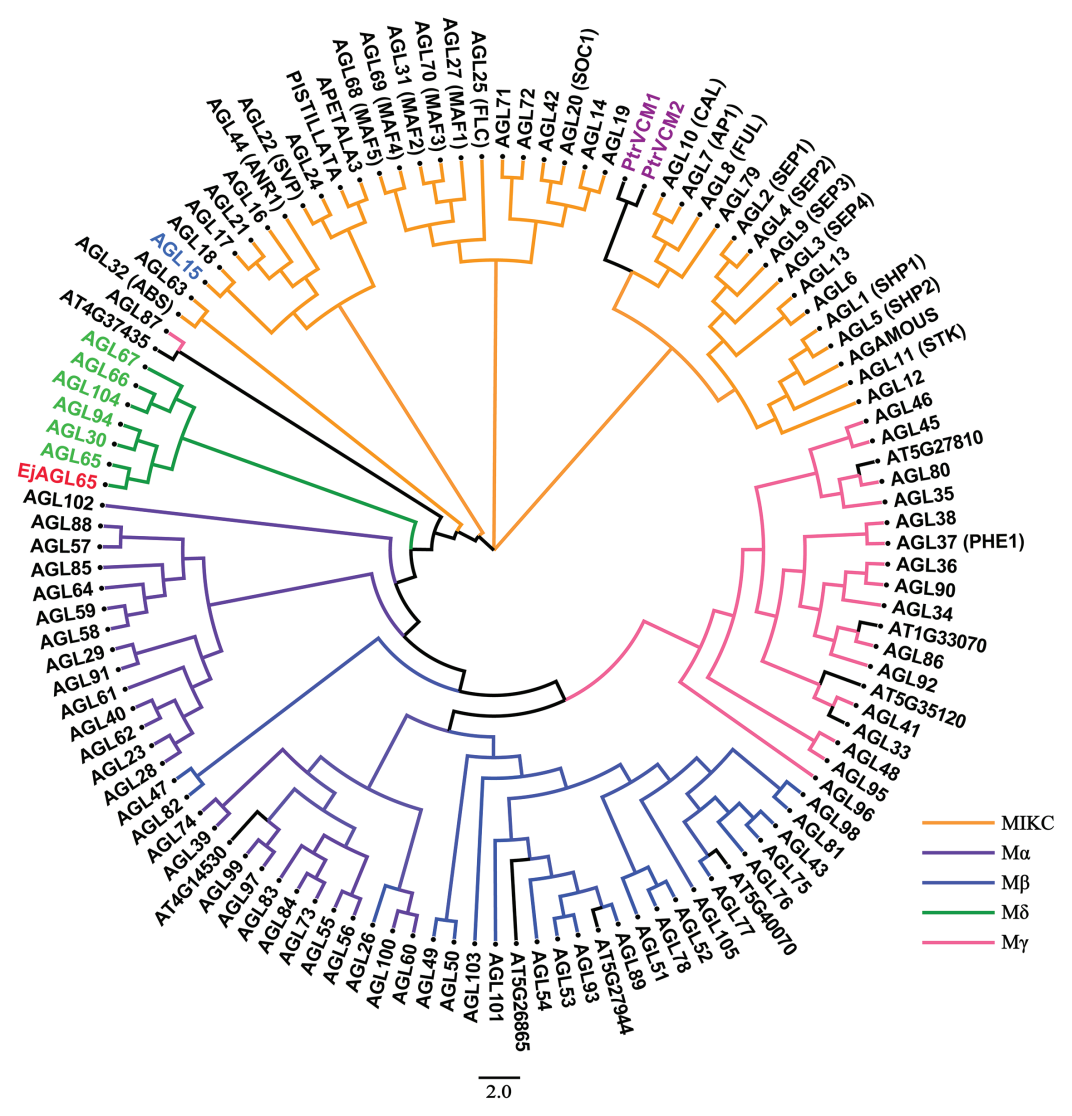
Phylogenetic analysis of EjAGL65 with 116 *Arabidopsis* MADS-box proteins and two Populus MADS-box proteins. The previously defined M*δ* group genes are shown in green. EjAGL65 was structurally closest to *Arabidopsis* AGL65 (At1g18750) and is shown in red. AGL15 (At5g13790), which was expressed in the lignified tissue of *Arabidopsis*, is shown in blue. PtrVCM1 and PtrVCM2, which affect vascular cambium proliferation activity, are in purple. The color of clades represents MIKC (orange), Mα (violet), Mβ (blue), Mδ (green), and Mγ (pink) subgroups of *Arabidopsis* MADS-box genes. Genes without annotations are labeled with an ID from the TAIR database.

Previous studies have shown that MADS-box genes are further classified into five subgroups, named MIKC, Mα, Mβ, Mγ, and Mδ, based on four conserved domains in *Arabidopsis* (Parenicová et al., 2003), and each group has a unique biological function. To predict the potential function of the MADS-box domain gene, we conducted a phylogenetic analysis. By comparing the amino acid sequence with MADS-box isoforms in *Arabidopsis*, we found that the *EjMYB8*-associated loquat MADS-box protein clustered with AtAGL65, AtAGL30, and AtAGL94, which are in the Mδ subgroup (Figure 2). Therefore, the newly identified MADS-box gene was named *EjAGL65*. AtAGL65, which is the putative ortholog of EjAGL65, forms a heterodimer with AtAGL104 and regulates pollen activity (Adamczyk and Fernandez, 2009). Besides, the MIKC group gene AtAGL15 regulates the class III peroxidase *PRX17* and the knockout line *prx17* showed reduced lignin content in the stem and siliques. The expression level of *PRX17* is downregulated in a 35S:AGL15 line, indicating the involvement of AtAGL15 in lignin metabolism (Cosio et al., 2017). However, EjAGL65 was not clustered with AtAGL15. These findings indicate that the EjAGL65 may not affect monolignol polymerization. The putative ortholog of EjAGL65 in *Arabidopsis* is not thought to be lignin-related.

### Association Between Lignin Accumulation and Expression of *EjAGL65*

**Figure 3:**
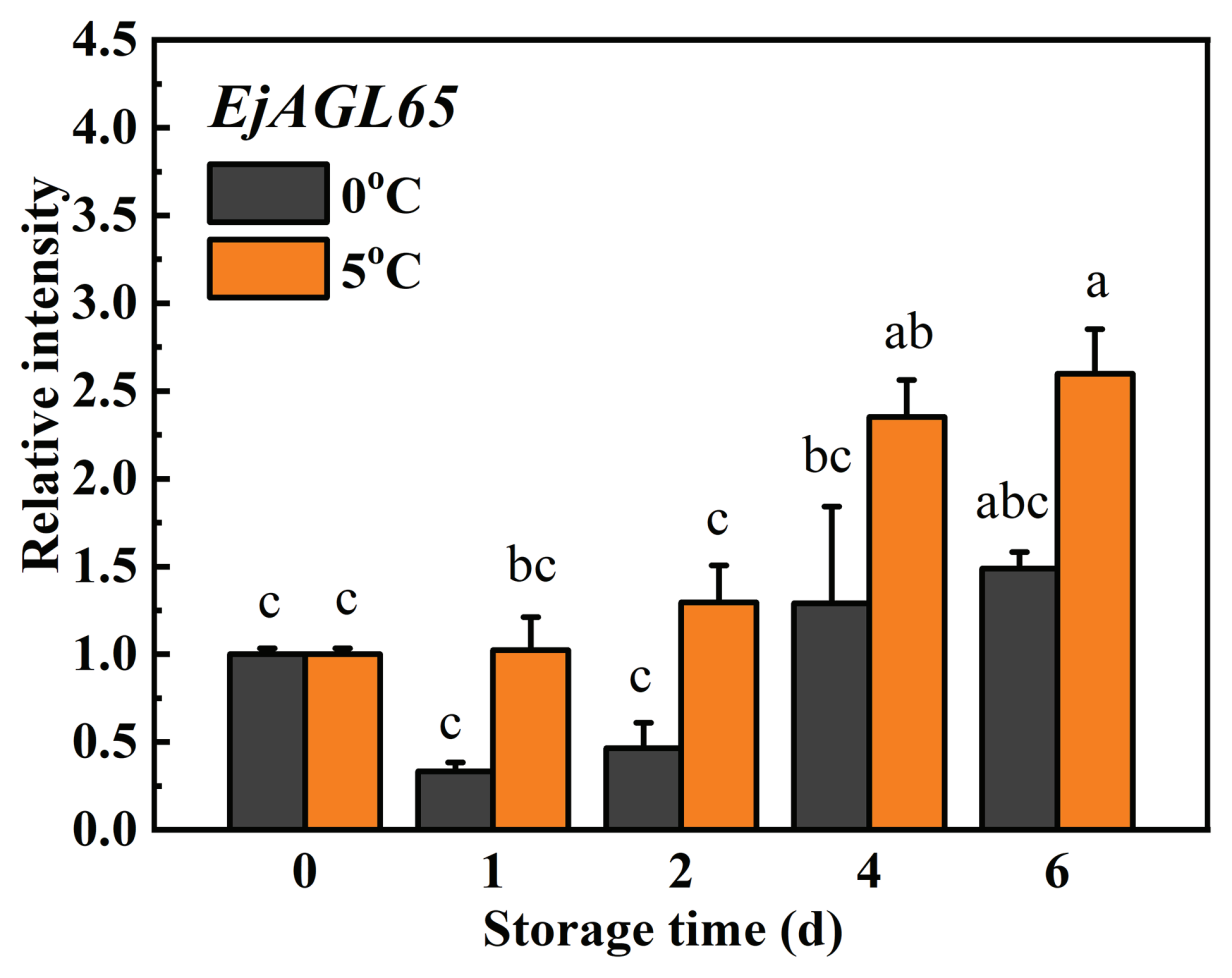
Expression of *EjAGL65* in response to temperature treatments at 0°C (control) and 5°C in “LYQ” loquat. Transcript levels of *EjAGL65* before the temperature treatment were set to 1. Error bars indicate the SE of three biological replicates. One-way ANOVA followed by Tukey’s comparison was performed at *p* = 0.05.

The expression of *EjAGL65* was investigated to determine whether it responds to chilling injury, which is critical evidence in support of it regulating chilling-induced lignification. Treatments at 0 and 5°C were routinely applied when characterizing candidate genes that relate to chilling injury of loquat fruits because 0°C induces lignification, while 5°C alleviates it (Cai et al., 2006). As shown in Figure 3, the average transcripts of *EjAGL65* decreased at 0°C on the first and second days, though the difference was not significant according to Turkey’s test. The transcription of *EjAGL65* did not decrease at 5°C during the first 2 days of treatment, followed by significant increases later. Based on its expression profile, *EjAGL65* was more sensitive to treatment at 0°C than 5°C. Xu et al. (2014b) tested fruit firmness and lignin content using the same batch of materials. The firmness and lignin content increased rapidly at 0 and 5°C during the first 2 days, which was opposite from the observed change of *EjAGL65* transcripts. This information indicates that *EjAGL65* is negatively correlated with changes in fruit firmness and lignin content. In addition, the decrease in *EjAGL65* transcripts stopped after 4 days of storage at 0°C, indicating an unknown mechanism preventing *EjAGL65* transcripts from continuously decreasing. *EjAGL65* is a candidate negative regulator of the cold-induced lignification process in loquat fruit flesh.

### Regulation of *EjAGL65* on Promoters of *EjMYB8* and Lignin Biosynthesis-Related Genes

**Figure 4:**
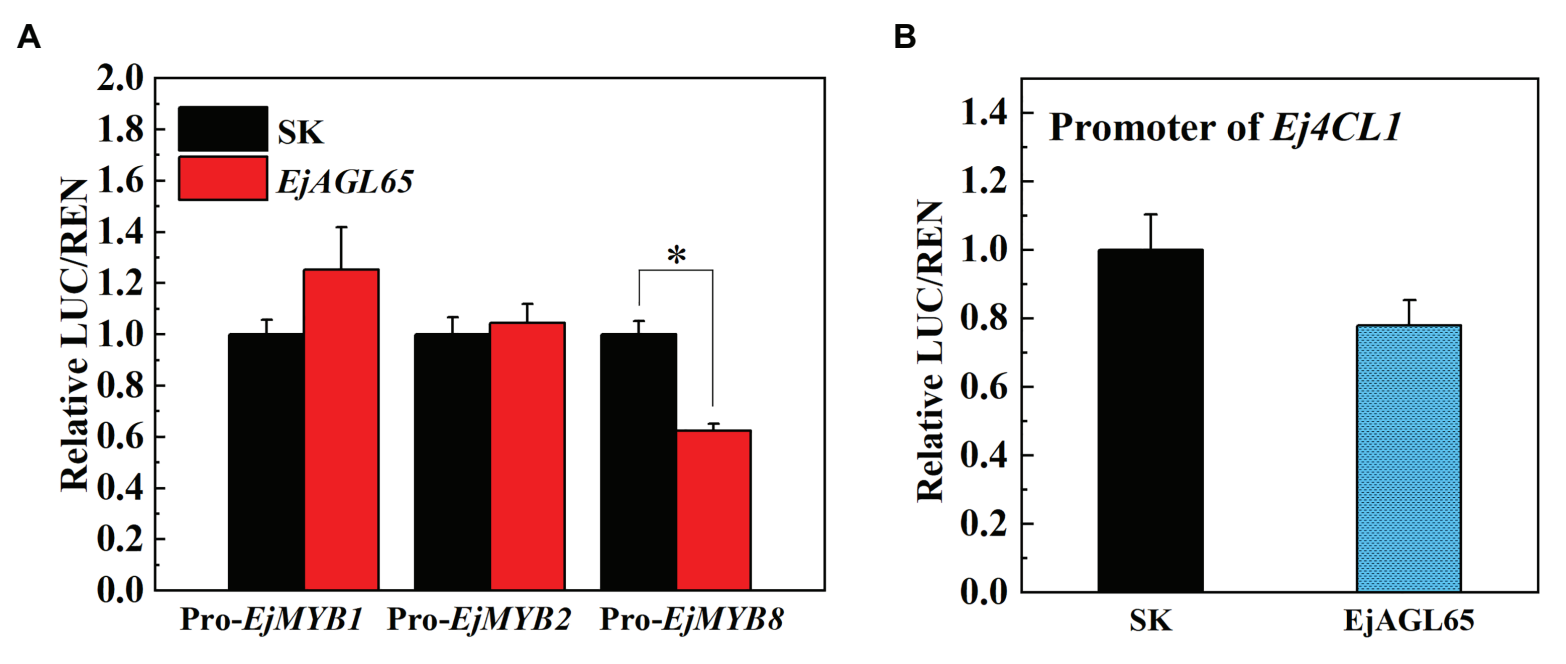
Luciferase assay between EjAGL65 and lignin biosynthesis-related genes. The SK vector contains the coding sequence of EjAGL65; the LUC vectors with the insertion of the *EjMYB1*, *EjMYB2*, *EjMYB8* **(A)**, and *Ej4CL1* **(B)** promoter were pairwise transformed into the *Agrobacterium* strain GV1301 followed by leaf infection. The LUC/REN value represents the activation (above 1) or inhibition (below 1) effect generated by EjAGL65. A combination of the Empty SK vector and LUC vectors with different promoters was used as a calibration (set as 1). Error bars represent five replicates. The asterisk indicates a significant difference according to the Student’s *t*-test at a significance level of 0.05 (^*^p < 0.05).

Possible regulatory actions of EjAGL65 were tested with the promoter of *EjMYB8* because of the direct binding confirmed in the yeast one-hybrid assay. The dual-luciferase assay showed that the activity of the *EjMYB8* promoter was significantly repressed after transient overexpression of *EjAGL65*. We also tested the promoter activity of *EjMYB1* and *EjMYB2*, another two EjMYB members related to lignin. However, the transient overexpression of EjAGL65 did not significantly change the promoter activity of *EjMYB1* and *EjMYB2* (Figure 4).

*Ej4CL1* is a key gene for loquat fruit lignification and the direct target of EjMYB8. The interaction between EjAGL65 and the promoter of *Ej4CL1* was also tested to further characterize the effect of EjAGL65 on the *Ej4CL1* promoter without EjMYB8. Protein-DNA interaction was analyzed using a yeast one-hybrid assay and dual-luciferase assay. Consequently, the activity of the *Ej4CL1* promoter was slightly but not significantly reduced due to the transient overexpression of EjAGL65 (Figure 4). In addition, EjAGL65 cannot bind the promoter of *Ej4CL1 in vivo* as shown by the yeast one-hybrid assay (Supplementary Figure S2), suggesting that EjAGL65 alone is not sufficient to affect the activity of the *Ej4CL1* promoter.

**Figure 5:**
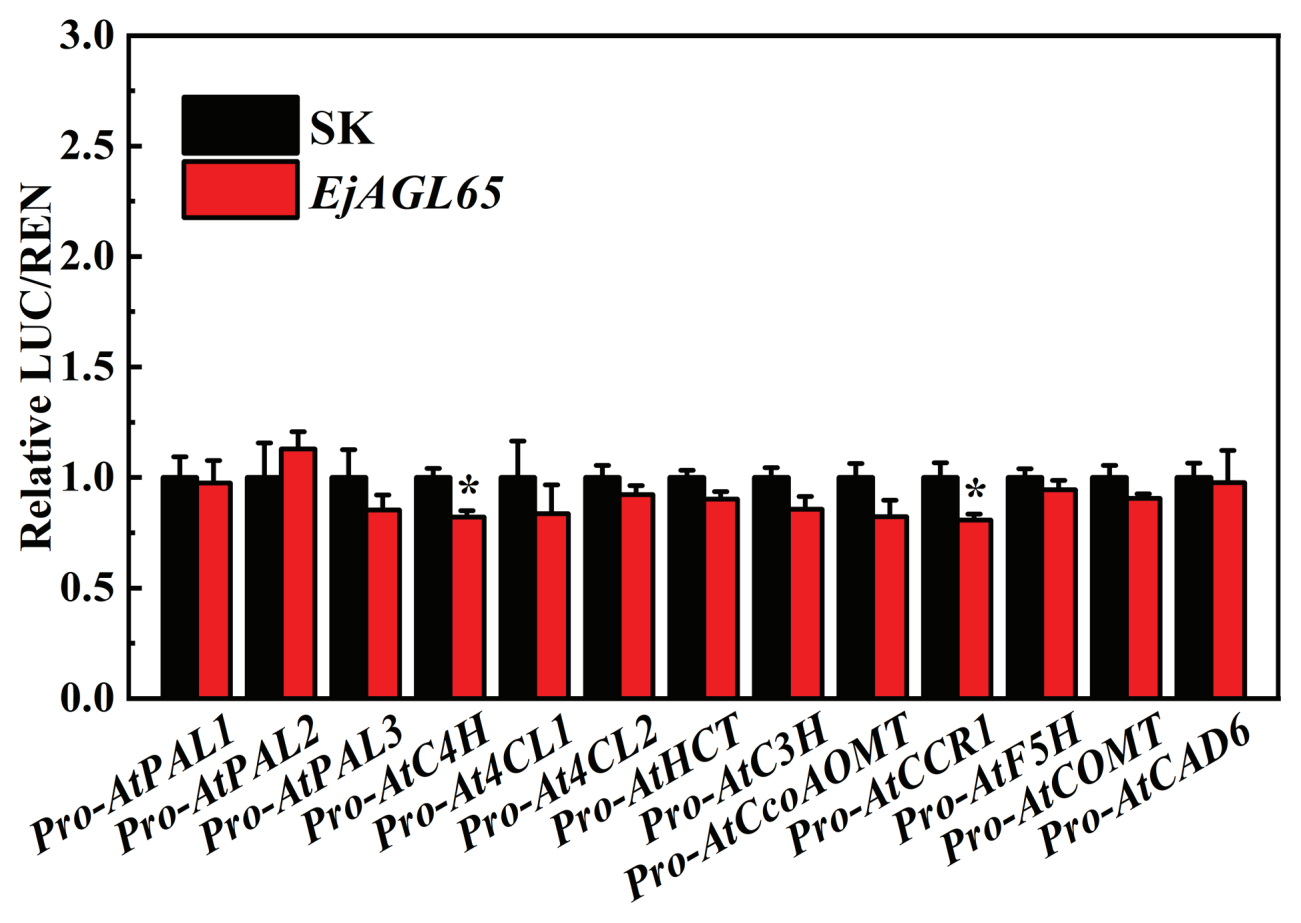
Interactions between *EjAGL65* and *Arabidopsis* genes involved in lignin biosynthesis. A combination of the empty SK vector and LUC vectors with different promoters was used as a calibration (set as 1). Error bars resulted from five replicates. The asterisk indicates that the relative LUC/REN value was significantly different between SK and EjAGL65 based on the Student’s *t*-test at a significance level of 0.05.

In addition, we chose the well-defined lignin biosynthesis-related genes from *Arabidopsis* to discover potential regulatory targets of EjAGL65. Consequently, most of the promoter activity of selected genes could not be regulated by EjAGL65. It is unclear whether the statistically significant activity against *AtC4H* and *AtCCR1* promoters (marked with asterisks in Figure 5) is sufficient to alter the biological function. Overall, the target preference of *EjAGL65* suggests it might selectively affect transcription factors, such as MYB family members, rather than structural genes of lignin biosynthesis.

## Discussion

### Confirmation of a MADS-Box Gene Manipulating Lignin Biosynthesis in Fruit Flesh

In recent years, knowledge on fruit flesh lignification has advanced partially due to experiments on protein-DNA interactions (Xu et al., 2015; Wang et al., 2016) that helped to unravel key aspects of the underlying regulatory network. Previous studies have demonstrated that *EjAP2-1* works in coordination with *EjMYB1* and *EjMYB2* for transcriptional regulation of *Ej4CL1* (Zeng et al., 2015). Zhang et al. (2020) recently found that the EjERF39 and EjMYB8 protein complex activates *Ej4CL1* by protein-DNA interaction with the promoter of *Ej4CL1*. Furthermore, utilization of the loquat transcriptome has allowed for the discovery of more lignification-related transcription factors (Lin et al., 2018; Liu et al., 2019). However, MADS-box genes are not frequently studied. In the presented study, we found the MADS-box domain gene *EjAGL65* using a yeast one-hybrid library screening assay. Data from a luciferase assay suggests that transient overexpression of EjAGL65 represses the transcription of *EjMYB8*, which is considered as a key activator of *Ej4CL1* (Wang et al., 2016), indicating the involvement of EjAGL65 in lignin biosynthesis. Interestingly, there is limited evidence of MADS-box genes being associated with lignin biosynthesis. Instead, MADS-box genes play vital roles in plant morphological development (Yu et al., 2014) and precise control of flowering and fruit development (Michaels and Amasino, 1999). AtAGL65, AtAGL30, and AtAGL94, which were determined to be the closest homologs of EjAGL65, control the maturation of pollen and pollen tube growth (Adamczyk and Fernandez, 2009). Tomato MADS-RIN is a primary ripening regulator that is highly expressed in fruit tissue (Vrebalov et al., 2002). The cell wall components were altered in the *rin* mutant, resulting in firmer texture, but the influence of RIN on lignin deposition was not clear (Li et al., 2018). Although the repression of another tomato MADS-box gene, SlTAGL1, results in lignin accumulation, the regulatory mechanism is unknown. Recently, a link between the MADS-box gene and lignin was established. *AtAGL15*, a MIKC type MADS-box gene, has been found to directly interact with the class III peroxidase PRX17, which causes ectopic lignin distribution in *Arabidopsis* stems if silenced (Cosio et al., 2017). Meanwhile, ectopic expression of *VCM1* and *VCM2*, two Populus MADS-box genes, leads to abnormal secondary growth by the regulation of the auxin transporter PIN5 (Zheng et al., 2020). By comparing amino acid sequences with *Arabidopsis* MADS-box genes, we found that AtAGL15, VCM1, and VCM2 belong to the MIKC group members. However, EjAGL65 lacks the K-box domain and is located in the Mδ subgroup (Supplementary Figure S3), indicating different mechanisms of lignin regulation between EjAGL65 and the other reported secondary cell wall related MADS-box genes. The Mδ subgroup consists of only six members in *Arabidopsis* and is crucial for pollen maturation (Verelst et al., 2007). The function of the Mδ genes is conserved in the land plants studied thus far (Kwantes et al., 2012), indicating their importance during plant evolution. The discovery of EjAGL65, which can alter lignin biosynthesis-related transcription factors, might suggest a novel function of the Mδ subgroup that is distinct from pollen development.

### Implications for the Regulation Mechanism Underlying the Expression Pattern of *EjAGL65* Transcripts

Several studies have been conducted on the expression of the MADS-box gene transcripts during different developmental stages (Qin et al., 2012; Niu et al., 2016). However, in fruit, the dynamic modulations of MADS-box genes in response to environmental stimuli, such as chilling, have rarely been studied. Here, transcript levels of *EjAGL65* were measured during the cold storage of loquat fruits. *EjAGL65* mRNA levels rapidly decreased during the first 48 h, but gradually recovered to their initial level, representing a response to chilling at days 1 and 2. Considering that the lignin content of loquat fruits also correlates with the pattern of *EjAGL65* transcripts, there may be specific mechanisms preventing the *EjAGL65* transcripts from continuing to decrease. Generally, genes responsible for cold perception have various means of preventing overexpression or insufficient expression at the transcriptional or translational level, such as SIZ1 and HOS1 proteins, which antagonistically determine the degradation of the ICE1 protein (Miura et al., 2007). Although, we cannot yet conclude whether *EjAGL65* is located in the cold signal transduction network, revealing such a feedback mechanism for precisely controlling the expression of *EjAGL65* would provide a better understanding of chilling induced flesh lignification.

### Lignin-Related *EjMYB8* Is Controlled by a MADS-Box Domain Protein

The relationship between MYB genes and lignin biosynthesis, as well as NAC master switches have been extensively studied in model plants, trees, and grasses. Lignin-related MYB genes are primarily regulated by NAC domain proteins at the transcription level in vascular tissue (Ohtani and Demura, 2019). The NAC-MYB based regulatory network is relatively conserved in the plant kingdom; it even exists in *Physcomitrella patens*, which is a moss that does not accumulate lignin (Xu et al., 2014a). Nevertheless, information about genes other than the NAC family that affect lignin-related MYB genes is still limited. Only the hAT transposase family gene *PtrhAT* was reported as the regulator of *PtrMYB021* (Xie et al., 2018). Several NAC genes have been characterized as activators of the lignification process in loquat flesh. However, no evidence indicates that NAC genes target lignin-related MYB genes, such as *EjMYB1*, whose homologs in *Arabidopsis* (*AtMYB58/63*) are regulated by the NAC-MYB transcription cascade (McCarthy et al., 2009; Zhou et al., 2009). On the contrary, the reported loquat NAC genes were confirmed as neither having a biological effect on MYB genes nor directly binding their promoter (Xu et al., 2015; Ge et al., 2017). These results suggest that the loquat may recruit new players for regulating stress induced lignification in flesh tissue. In this study, we performed a library-scale screening to determine the upstream regulators of *EjMYB8*. Nevertheless, no NAC genes were found in the library pool after *in vivo* screening in yeast. Interestingly, a MADS-box domain protein, EjAGL65, physically binds to the promoter of *EjMYB8*, forming the MADS-MYB cascade. The results provide additional information about how to unravel a new hub in the complicated regulatory network of fruit flesh lignin biosynthesis.

### Multiple Transcription Factors Constitute the Complicated Regulatory Network Based on *Ej4CL1*

Research on transcriptional regulation in the lignification of loquat flesh began with identifying two MYB family genes, *EjMYB1* and *EjMYB2* (Xu et al., 2014b). Subsequently, EjAP2-1 was found to be involved in the regulatory network by showing protein-protein interactions with either *EjMYB1* or *EjMYB2* (Zeng et al., 2015). With the further screening of MYB genes, *EjMYB8* was proposed as an effective activator of lignin biosynthesis (Wang et al., 2016). The activation effect of *EjMYB8* could be further enhanced by forming a protein complex with *EjERF39* (Zhang et al., 2020), but there was limited information about negative regulators of *EjMYB8*. These MYB genes share the same target, located in the promoter region of *Ej4CL1*, a gene that plays an essential role in the biosynthesis of lignin monomers (Li et al., 2017), which complicates the mechanism by which *Ej4CL1* transcripts are adjusted.

**Figure 6:**
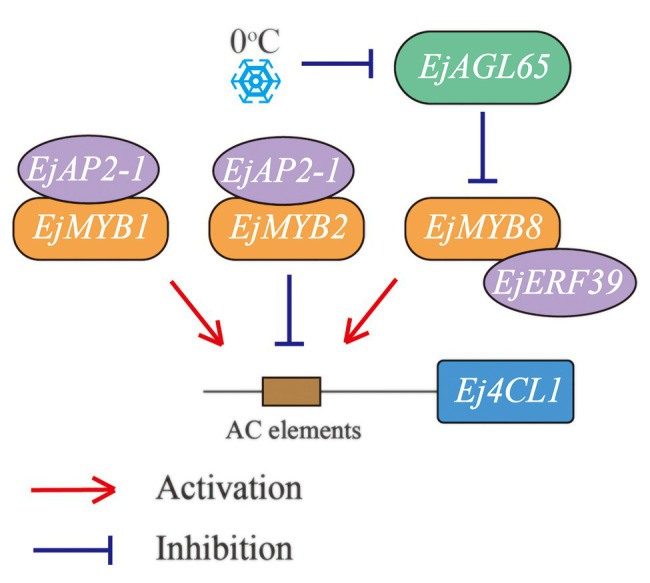
Regulatory routes identified based on *Ej4CL1*. Three MYB genes are physically associated with *Ej4CL1*, acting as mediators for binding with other transcription factors. Both EjMYB1 and EjMYB2 can recruit EjAP2-1 to repress their transcriptional activities. EjMYB8 is controlled at both protein level (by EjERF39) and mRNA level (by EjAGL65). Lines with an arrow or a dashed terminal indicate activation or repression, respectively. Two genes shown in a sequence indicate a confirmed protein-protein interaction with each other.

The present study showed that the MADS-box gene *EjAGL65* affects loquat flesh lignin accumulation *via* repressing the transcription of the characterized lignin activator *EjMYB8* (Figure 6). *EjAGL65* transcripts showed a temperature-dependent pattern similar to its *Arabidopsis* homologs, providing a new hub for characterizing a chilling induced regulation network of flesh lignification. The system contains three MYB genes, two members of the AP2/ERF gene family, and a MADS-box gene, forming three regulatory pathways. Interestingly, only two of the abovementioned genes negatively regulate lignin biosynthesis at a transcription level, while the others either have identical activation effects or function through protein-protein interaction. Identifying additional factors and understanding the synergistic or antagonistic effects among the four pathways of the network will be the main focus of future research.

## Conclusion

The present study discovered a chilling-repressed MADS-box gene named *EjAGL65*. Results of dual luciferase and yeast one-hybrid assays indicated that *EjAGL65* transcriptionally inhibits the promoter of *EjMYB8*, forming a MADS-MYB transcriptional cascade. The homologs of EjAGL65 were not known to affect the lignin biosynthesis pathway in model plants, revealing a novel route for the regulation of stress induced lignin.

## Data Availability Statement

The datasets presented in this study can be found in online repositories. The names of the repository/repositories and accession number(s) can be found in the article/Supplementary Material.

## Author Contributions

KC conceived the experiments. HG and MZ conducted the study and processed the data. HG and YS wrote the manuscript. KC, XL, and XY revised the manuscript. All authors contributed to the article and approved the submitted version.

### Conflict of Interest

The authors declare that the research was conducted in the absence of any commercial or financial relationships that could be construed as a potential conflict of interest.

## Footnotes

^1^https://www.ncbi.nlm.nih.gov/

^2^https://www.arabidopsis.org/

^3^http://tree.bio.ed.ac.uk/software/figtree/

^4^http://bioinfo.ut.ee/primer3/
