# Supplementary figures and images for: The MADS-Box Transcription Factor EjAGL65 Controls Loquat Flesh Lignification via Direct Transcriptional Inhibition of EjMYB8
Source: Front Plant Sci. 2021 Apr 7;12:652959. doi: 10.3389/fpls.2021.652959 (PMC8058365; doi:10.3389/fpls.2021.652959)

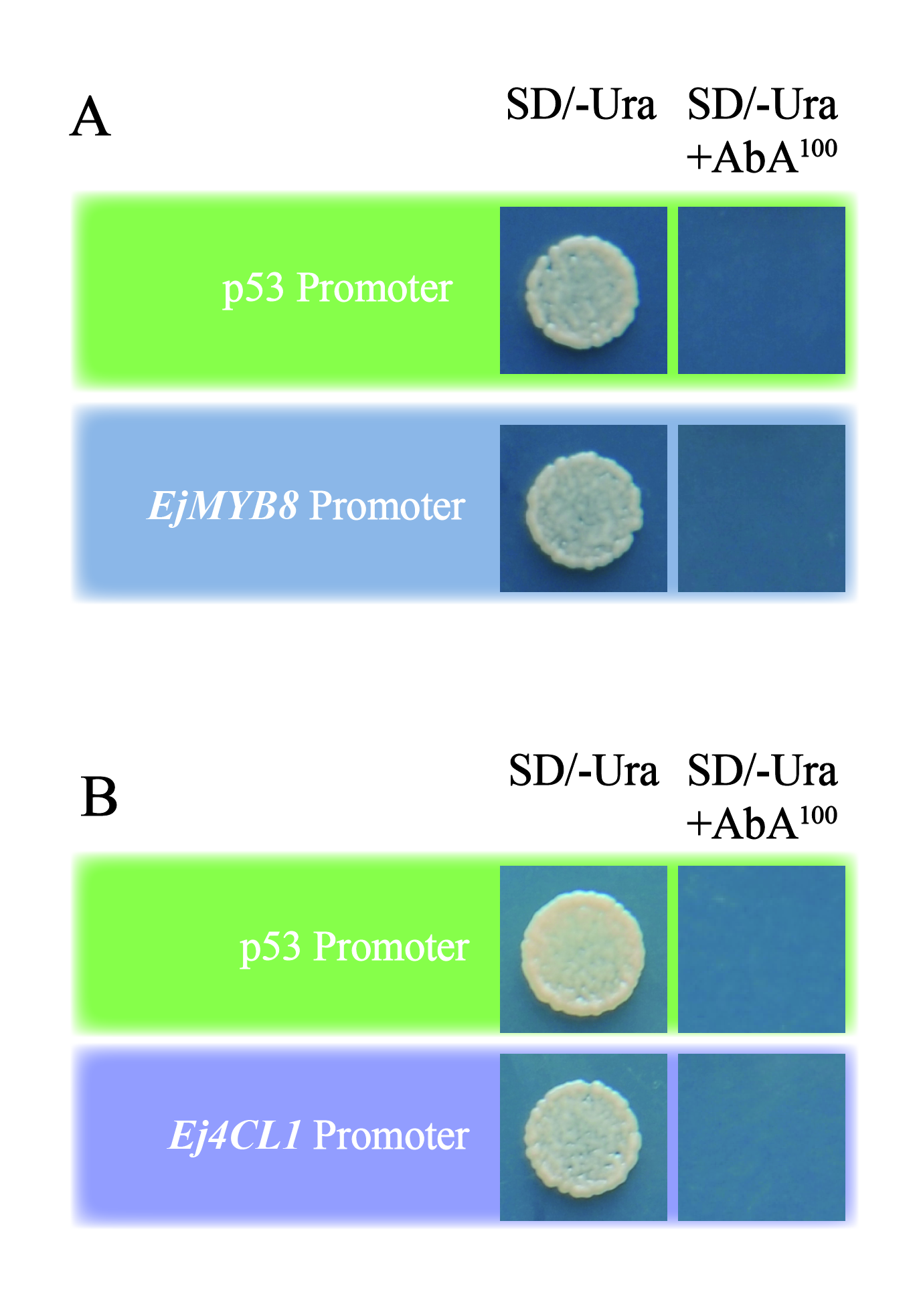

Supplement: Supplementary file 1 [file Image_1.TIF]

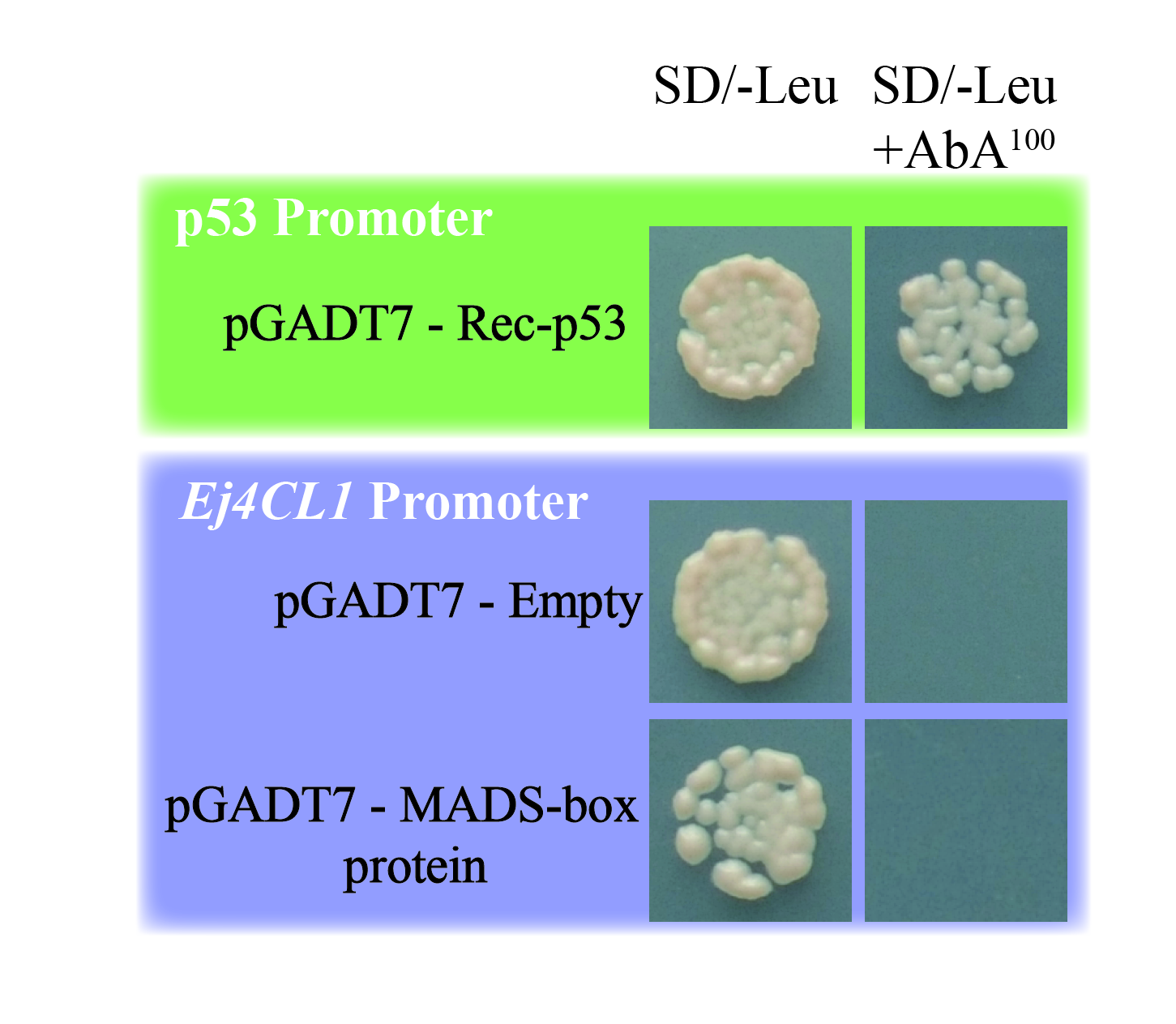

Supplement: Supplementary file 2 [file Image_2.TIF]

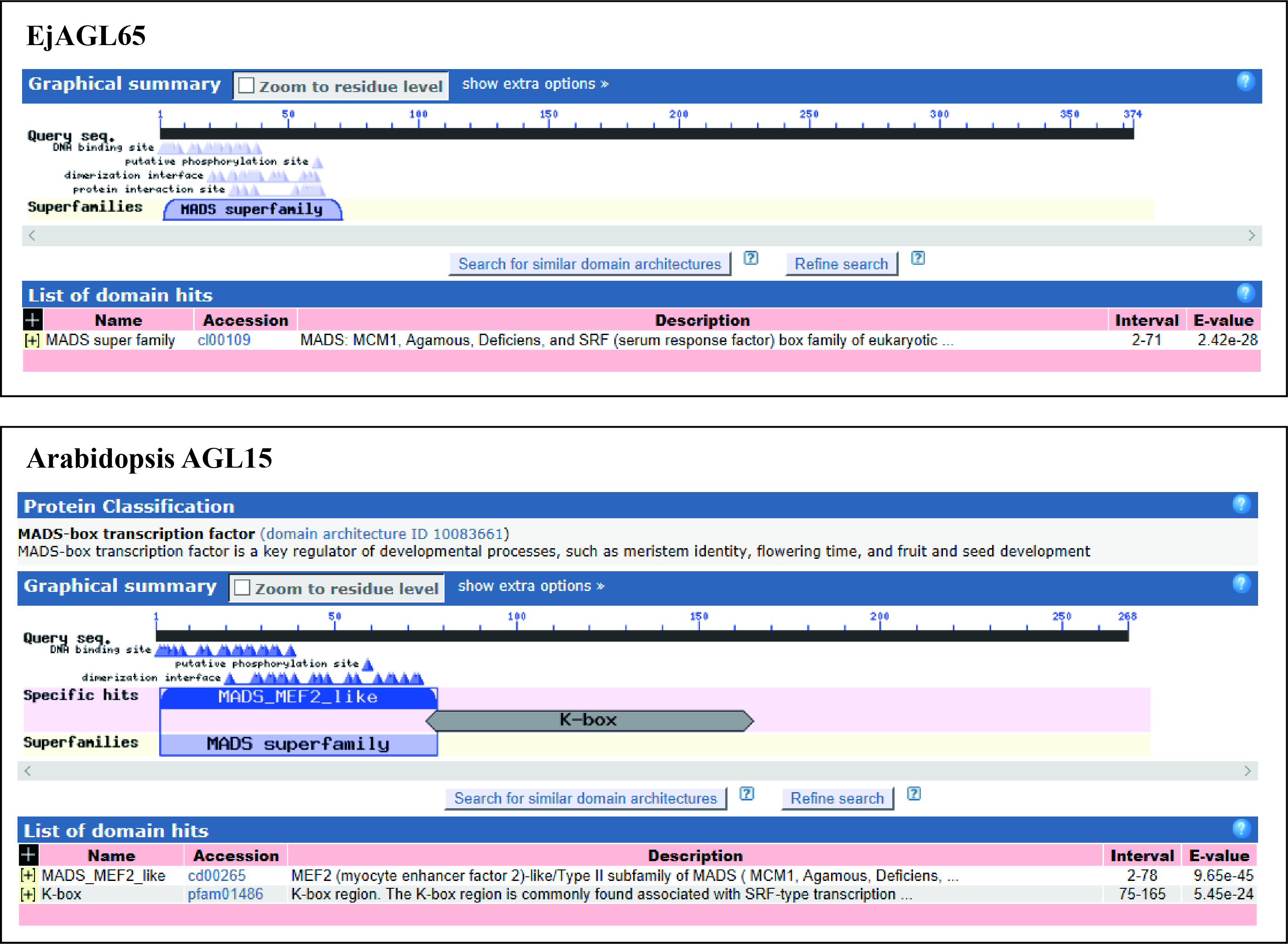

Supplement: Supplementary file 3 [file Image_3.TIF]
