# Supplementary material for: The MADS-Box Transcription Factor EjAGL65 Controls Loquat Flesh Lignification via Direct Transcriptional Inhibition of EjMYB8
Source: Front Plant Sci. 2021 Apr 7;12:652959. doi: 10.3389/fpls.2021.652959 (PMC8058365; doi:10.3389/fpls.2021.652959)

# *Supplementary Material*

**Table S1** Primers for *EjMYB8* promoter isolation by GenomeWalker$^{TM}$ universal kit (Clontech)

| Gene | Gene-specific primer (5' to 3') | Nested gene-specific primer (5' to 3') |
|---|---|---|
| *EjMYB8* | CTGGCCTCAAGTAGTTTGTCCATCTG | TGGCCATGCTGCTGAATGTAAGTGAC |

**Table S2** Primers used for pAbAi vector construction

| Gene | Vector | Forward primer (5' to 3') | Reverse primer (5' to 3') |
|---|---|---|---|
| *EjMYB8* | pAbAi | TCGAGCTCCTGGTGGGCTGTATTTTAATATTAATGAATGTATAAC | AGGTCGACTTTGATCAGAAAGTGATTAAAAACTCTCTGTTC |

**Table S3** Transcriptional factor candidates found in Y1H Library screening assay, which are chosen for additional confirmation.

| Gene annotations | Genbank No. | Counts |
|---|---|---|
| Pyrus x bretschneideri CBS domain-containing protein CBSX3 | XM_009380800.1 | 1 |
| Pyrus x bretschneideri MADS-box protein SVP-like | XM_009371005.1 | 1 |
| Pyrus x bretschneideri histone H3-like centromeric cnp1 | XM_009346944.1 | 1 |
| Pyrus x bretschneideri U-box domain-containing protein | XM_009363371.1 | 1 |
| Pyrus x bretschneideri auxin-responsive protein SAUR40-like | XM_009378552.2 | 1 |

**Table S4** Primers used for homologous cloning. Genes were given temporary names according to their annotations.

| Genes | Forward primer (5' to 3') | Reverse primer (5' to 3') |
|---|---|---|
| CBS domain-containing protein | ATGCAAGGAGTACTCAAAAC | CTAGTAACCACCTTGAATAA |
| MADS-box protein SVP-like | ATGGGAAGGGTTAAGTTAAA | CTACAATCTCACGTTTGGTT |
| histone H3-like centromeric protein | ATGGCGAGGATTAAGCACAC | CTAGTGTATATTGCGGCTAA |
| U-box domain-containing protein | ATGTCGTCCGTGGCCGTGGC | TTAAGTCAAACCTGACCTCC |
| auxin-responsive protein SAUR40-like | ATGTCTACGGTGAAGAACTG | CTAGGATCCTTGTACAACTG |

**Table S5** Primers used for constructions of pGADT7 vectors.

| Annotations | Forward primer (5' to 3') | Reverse primer (5' to 3') |
|---|---|---|
| CBS domain-containing protein CBSX3 | GAGGCCAGTGAATTCATGCAAGGAGTACTCAAAAC | GAGCTCGATGGATCCCTAGTAACCACCTTGAATAA |
| MADS-box protein SVP-like | GAGGCCAGTGAATTCATGGGAAGGGTTAAGTTAAAG | GAGCTCGATGGATCCCTACAATCTCACGTTTGGTTG |
| histone H3-like centromeric protein | GAGGCCAGTGAATTCATGGCGAGGATTAAGCACAC | GAGCTCGATGGATCCTCAGCAGAACCTCCCTTCCC |
| auxin-responsive protein SAUR41-like | GAGGCCAGTGAATTCATGTCTACGGTGAAGAACTG | GAGCTCGATATCGATCTAGGATCCTTGTACAACTG |
| U-box domain-containing protein | GAGGCCAGTGAATTCATGTCGTCCGTGGCCGTGGC | GAGCTCGATGGATCCTTAAGTCAAACCTGACCTCC |

**Table S6** Genbank number of 5 sequences acquired by homologous cloning.

| Genes | Genbank No. |
|---|---|
| CBS domain-containing protein | MF942413.1 |
| MADS-box protein SVP-like | MF942415.1 |
| histone H3-like centromeric protein | MT362564 |
| U-box domain-containing protein | MT362570 |
| auxin-responsive protein SAUR40-like | MT362567 |

**Table S7** The names and IDs of genes used in the phylogenetic analysis. Genes without a formal name are labeled with an ID from the TAIR database.

| No | Gene names | IDs |
|---|---|---|
| 1 | AGL1 | AT3G58780 |
| 2 | AGL2 | AT5G15800 |
| 3 | AGL3 | AT2G03710 |
| 4 | AGL4 | AT3G02310 |
| 5 | AGL5 | AT2G42830 |
| 6 | AGL6 | AT2G45650 |
| 7 | AGL7 | AT1G69120 |
| 8 | AGL8 | AT5G60910 |
| 9 | AGL9 | AT1G24260 |
| 10 | AGL10 | AT1G26310 |
| 11 | AGL11 | AT4G09960 |
| 12 | AGL12 | AT1G71692 |
| 13 | AGL13 | AT3G61120 |
| 14 | AGL14 | AT4G11880 |
| 15 | AGL15 | AT5G13790 |
| 16 | AGL16 | AT3G57230 |
| 17 | AGL17 | AT2G22630 |
| 18 | AGL18 | AT3G57390 |
| 19 | AGL19 | AT4G22950 |
| 20 | AGL20 | AT2G45660 |
| 21 | AGL21 | AT4G37940 |
| 22 | AGL22 | AT2G22540 |
| 23 | AGL23 | AT1G65360 |

| | | |
|---|---|---|
| 24 | AGL24 | AT4G24540 |
| 25 | AGL25 | AT5G10140 |
| 26 | AGL26 | AT5G26880 |
| 27 | AGL27 | AT1G77080 |
| 28 | AGL28 | AT1G01530 |
| 29 | AGL29 | AT2G34440 |
| 30 | AGL30 | AT2G03060 |
| 31 | AGL31 | AT5G65050 |
| 32 | AGL32 | AT5G23260 |
| 33 | AGL33 | AT2G26320 |
| 34 | AGL34 | AT5G26580 |
| 35 | AGL35 | AT5G26630 |
| 36 | AGL36 | AT5G26650 |
| 37 | AGL37 | AT1G65330 |
| 38 | AGL38 | AT1G65300 |
| 39 | AGL39 | AT5G27130 |
| 40 | AGL40 | AT4G36590 |
| 41 | AGL41 | AT2G26880 |
| 42 | AGL42 | AT5G62165 |
| 43 | AGL43 | AT5G40220 |
| 44 | AGL44 | AT2G14210 |
| 45 | AGL45 | AT3G05860 |
| 46 | AGL46 | AT2G28700 |
| 47 | AGL47 | AT5G55690 |
| 48 | AGL48 | AT2G40210 |
| 49 | AGL49 | AT1G60040 |
| 50 | AGL50 | AT1G59810 |
| 51 | AGL51 | AT4G02235 |
| 52 | AGL52 | AT4G11250 |
| 53 | AGL53 | AT5G27070 |
| 54 | AGL54 | AT5G27090 |
| 55 | AGL55 | AT1G60920 |
| 56 | AGL56 | AT1G60880 |
| 57 | AGL57 | AT3G04100 |
| 58 | AGL58 | AT1G28450 |
| 59 | AGL59 | AT1G28460 |
| 60 | AGL60 | AT1G72350 |
| 61 | AGL61 | AT2G24840 |
| 62 | AGL62 | AT5G60440 |
| 63 | AGL63 | AT1G31140 |
| 64 | AGL64 | AT1G29962 |
| 65 | AGL65 | AT1G18750 |
| 66 | AGL66 | AT1G77980 |
| 67 | AGL67 | AT1G77950 |
| 68 | AGL68 | AT5G65080 |
| 69 | AGL69 | AT5G65070 |
| 70 | AGL70 | AT5G65060 |

| | | |
|---|---|---|
| 71 | AGL71 | AT5G51870 |
| 72 | AGL72 | AT5G51860 |
| 73 | AGL73 | AT5G38620 |
| 74 | AGL74 | AT1G48150 |
| 75 | AGL75 | AT5G41200 |
| 76 | AGL76 | AT5G40120 |
| 77 | AGL77 | AT5G38740 |
| 78 | AGL78 | AT5G65330 |
| 79 | AGL79 | AT3G30260 |
| 80 | AGL80 | AT5G48670 |
| 81 | AGL81 | AT5G39750 |
| 82 | AGL82 | AT5G58890 |
| 83 | AGL83 | AT5G49490 |
| 84 | AGL84 | AT5G49420 |
| 85 | AGL85 | AT1G54760 |
| 86 | AGL86 | AT1G31630 |
| 87 | AGL87 | AT1G22590 |
| 88 | AGL88 | AT2G11990 |
| 89 | AGL89 | AT5G27580 |
| 90 | AGL90 | AT5G27960 |
| 91 | AGL91 | AT3G66656 |
| 92 | AGL92 | AT1G31640 |
| 93 | AGL93 | AT5G26950 |
| 94 | AGL94 | AT1G69540 |
| 95 | AGL95 | AT2G15660 |
| 96 | AGL96 | AT5G06500 |
| 97 | AGL97 | AT1G46408 |
| 98 | AGL98 | AT5G39810 |
| 99 | AGL99 | AT5G04640 |
| 100 | AGL100 | AT1G17310 |
| 101 | AGL101 | AT5G27050 |
| 102 | AGL102 | AT1G47760 |
| 103 | AGL103 | AT3G18650 |
| 104 | AGL104 | AT1G22130 |
| 105 | AGL105 | AT5G37415 |
| 106 | APETALA3 | AT3G54340 |
| 107 | PISTILLATA | AT5G20240 |
| 108 | AGAMOUS | AT4G18960 |
| 109 | AT1G33070 | AT1G33070 |
| 110 | AT4G14530 | AT4G14530 |
| 111 | AT4G37435 | AT4G37435 |
| 112 | AT5G26865 | AT5G26865 |
| 113 | AT5G27810 | AT5G27810 |
| 114 | AT5G27944 | AT5G27944 |
| 115 | AT5G35120 | AT5G35120 |
| 116 | AT5G40070 | AT5G40070 |
| 117 | EjAGL65 | MF942415.1 |
| 118 | PtrVCM1 | XM024582264.1 |

| | | |
|---|---|---|
| 119 | PtrVCM2 | XM002321675.3 |

**Table S8** Primer for Real-time PCR analysis

| Gene | Forward primer (5' to 3') | Reverse primer (5' to 3') |
|---|---|---|
| *EjAGL65* | CGTACCAGCAAATCCAGTGG | GCGAAGAAAGCCAAGCATGA |

**Table S9** Primers used for dual-luciferase assay

| Gene | Vector | Forward primer (5' to 3') | Reverse primer (5' to 3') |
|---|---|---|---|
| *EjAGL65* | pGreen II 0029 62-SK | GGGCTGCAGGAATTC ATGGGAAGGGTTAAGTTAAAG | CCCCTCGAGGTCGACCTACAATCT CACGTTTGGTTG |

**Table S10** The BLAST analysis of cDNA fragments that obtained from Y1H library screening. Annotation came from the NCBI database.

| Gene annotations | Genbank No. | Counts |
|---|---|---|
| Pyrus x bretschneideri ranBP2-type zinc finger protein | XM_009373999.1 | 1 |
| Pyrus x bretschneideri uncharacterized transcript variant X1 | XM_009344289.1 | 1 |
| PREDICTED: Pyrus x bretschneideri auxin-responsive protein SAUR40-like (LOC103965486), mRNA | XM_009378552.2 | 1 |
| Pyrus x bretschneideri plasma membrane ATPase 4 transcript variant X1 | XM_009340549.1 | 1 |

| | | |
|---|---|---|
| Pyrus x bretschneideri uncharacterized transcript variant X2 | XM_009344225.1 | 1 |
| Pyrus x bretschneideri CBS domain-containing CBSX3 | XM_009380800.1 | 1 |
| Pyrus x bretschneideri cactin-like transcript variant X1 | XM_009377370.1 | 1 |
| Pyrus x bretschneideri eukaryotic translation initiation factor 5-like transcript variant X2 | XM_009373548.1 | 1 |
| Pyrus x bretschneideri E3 SUMO-protein ligase SIZ1-like transcript variant X2 | XM_009344074.1 | 1 |
| Pyrus x bretschneideri LIMR family protein At5g01460 | XM_009379490.1 | 1 |
| Pyrus x bretschneideri protein decapping 5-like transcript variant X2 | XM_009357830.1 | 1 |
| Pyrus x bretschneideri protein WHI3-like transcript variant X1 | XM_009369975.1 | 1 |
| Pyrus x bretschneideri methyltransferase PMT21 transcript variant X2 | XM_009367471.1 | 1 |
| Pyrus x bretschneideri protein zinc induced faciliator-like transcript variant X2 | XM_009356801.1 | 1 |
| Pyrus x bretschneideri polyamine transporter At1g31830 transcript variant X2 | XM_009357351.1 | 1 |
| Pyrus x bretschneideri U-box domain-containing protein | XM_009363371.1 | 1 |
| Pyrus x bretschneideri uncharacterized transcript variant X3 | XR_665403.1 | 1 |
| Pyrus x bretschneideri E3 ubiquitin-protein ligase UPL6 transcript variant X1 | XM_009373803.1 | 1 |
| Pyrus x bretschneideri MADS-box protein SVP-like | XM_009371005.1 | 1 |
| Pyrus x bretschneideri LEC14B homolog | XM_009353905.1 | 1 |
| Pyrus x bretschneideri BTB/POZ domain-containing transcript variant X3 | XM_009376725.1 | 1 |

| | | |
|---|---|---|
| Malus x domestica uncharacterized transcript variant X3 | XR_528925.1 | 1 |
| Pyrus x bretschneideri mediator of RNA polymerase II transcription subunit 31 | XM_009365465.1 | 1 |
| Pyrus x bretschneideri histone H3-like centromeric protein cnp1 | XM_009346944.1 | 1 |
| Pyrus x bretschneideri inositol oxygenase 1-like transcript variant X2 | XM_009339646.1 | 1 |
| Pyrus x bretschneideri heat shock cognate 70 kDa transcript variant X1 | XM_009368431.1 | 1 |
| Pyrus x bretschneideri uncharacterized transcript variant X2 | XM_009340430.1 | 1 |
| Pyrus x bretschneideri mediator of RNA polymerase II transcription subunit | XM_009350452.1 | 1 |
| Pyrus x bretschneideri ectonucleotide pyrophosphatase/phosphodiesterase family member 3-like transcript variant X2 | XR_666796.1 | 1 |
| Pyrus x bretschneideri lysine-specific demethylase REF6-like | XM_009361233.1 | 1 |
| Pyrus x bretschneideri uncharacterized protein | XM_009373188.1 | 1 |
| Pyrus x bretschneideri uncharacterized WD repeat-containing protein | XM_009380840.1 | 1 |

**Figure S1** Auto-activation test of *EjMYB8* (A) and *Ej4CL1* (B) promoters. Y1HGold strain with the promoter of *EjMYB8* and *Ej4CL1* integrated into its genome was cultured on the SD/-Ura medium with Aureobasidin. The promoter of p53 was used as a negative control.

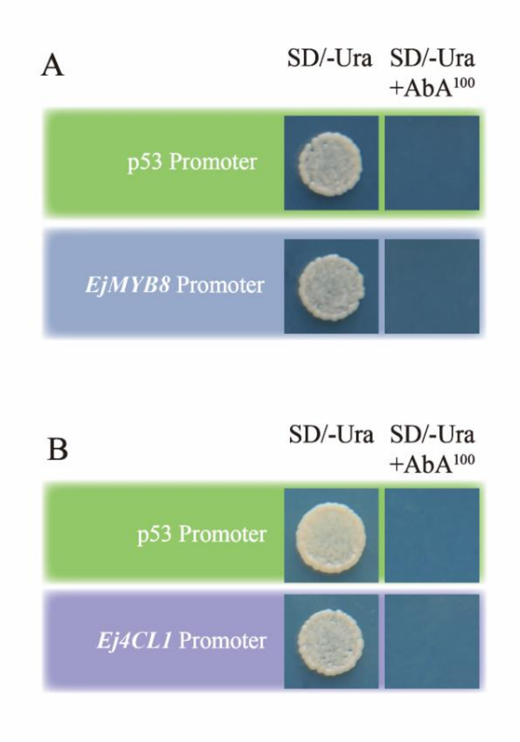


**Figure S2** Yeast one-hybrid assay for identifying the interaction between EjAGL65 and promoter of *Ej4CL1*. The promoter of p53 was used as a negative control.

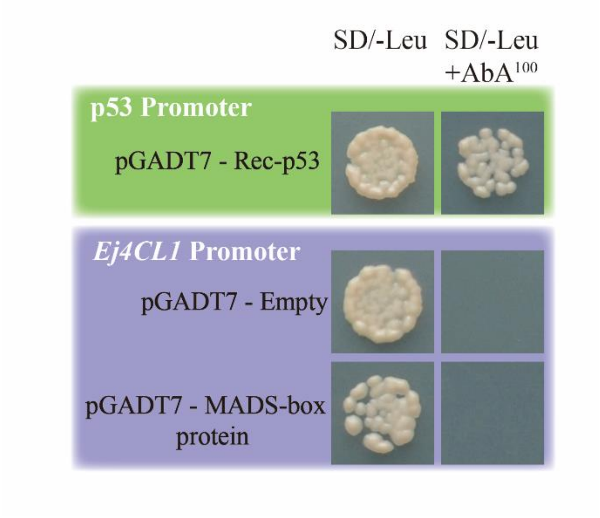

**Figure S3** The predicted conserve domain of EjAGL65 (Mδ subgroup) and Arabidopsis AGL15 (MIKC subgroup) by NCBI's conserved domain database.

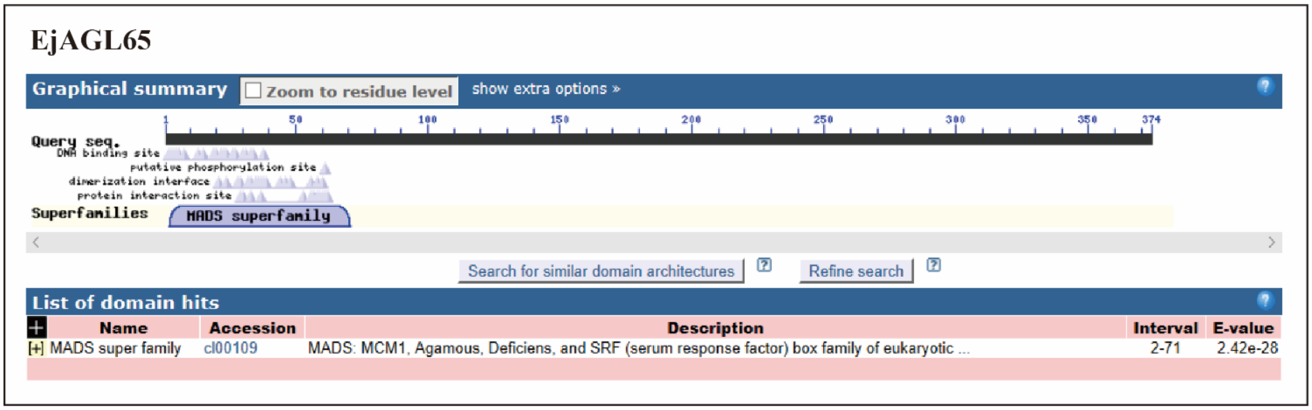


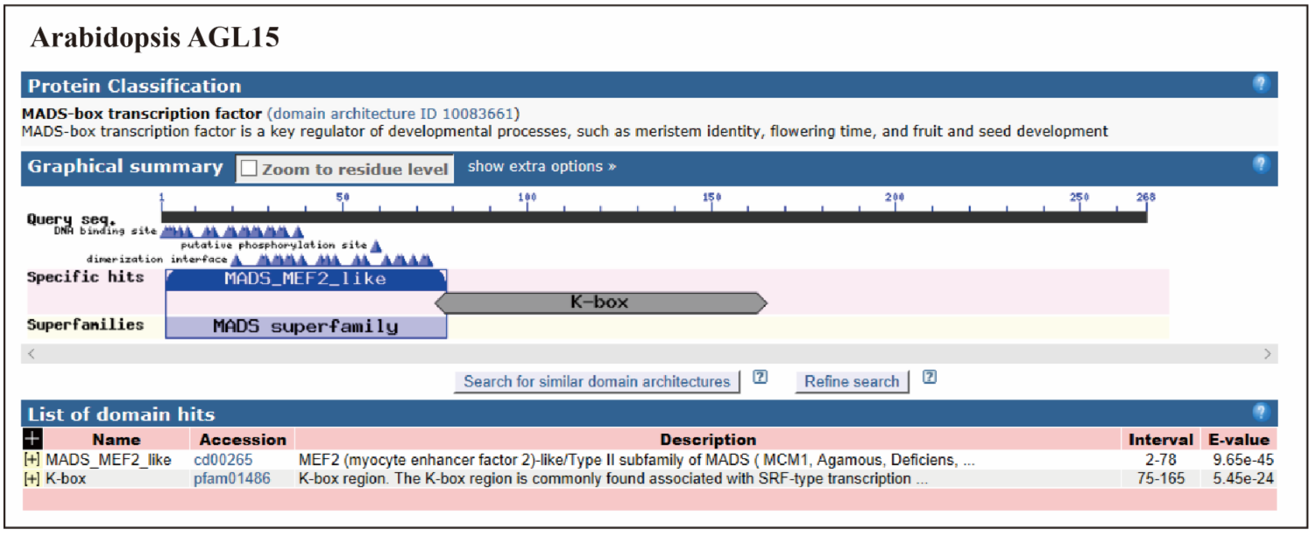

Supplement: Supplementary file 4 [file Data_Sheet_1.PDF]
